# Objective analysis of the topological organization of the human cortical visual connectome suggests three visual pathways

**DOI:** 10.1016/j.cortex.2017.03.020

**Published:** 2018-01

**Authors:** Koen V. Haak, Christian F. Beckmann

**Affiliations:** a Donders Institute for Brain, Cognition and Behaviour, Centre for Cognitive Neuroimaging, Radboud University, Nijmegen, The Netherlands; b Department of Cognitive Neuroscience, Radboud University Medical Centre, Nijmegen, The Netherlands; c Oxford Centre for Functional Magnetic Resonance Imaging of the Brain (FMRIB), University of Oxford, Oxford, United Kingdom

**Keywords:** Visual cortex, Processing pathways, Functional connectivity, Resting-state fMRI, Human connectome project

## Abstract

The cortical visual system is composed of many areas serving various visual functions. In non-human primates, these are broadly organised into two distinct processing pathways: a ventral pathway for object recognition, and a dorsal pathway for action. In humans, recent theoretical proposals suggest the possible existence of additional pathways, but direct empirical evidence has yet to be presented. Here, we estimated the connectivity patterns between 22 human visual areas using resting-state functional MRI data of 470 individuals, leveraging the unprecedented data quantity and quality of the Human Connectome Project and a novel probabilistic atlas. An objective, data-driven analysis into the topological organisation of connectivity and subsequent quantitative confirmation revealed a highly significant triple dissociation between the retinotopic areas on the dorsal, ventral and lateral surfaces of the human occipital lobe. This suggests that the functional organisation of the human visual system involves not two but three cortical pathways.

## 1 Introduction

Human visual cortex is composed of many visual areas, each of which is known to contain a map of the visual field and can be linked to various visual functions (Wandell, Dumoulin, & Brewer, 2007). The overall organisation of information processing defined by the connections between these visual field maps is less well understood. The prevailing view, first introduced in the early eighties based on disconnection studies in the macaque (MISHKIN et al., 1983, UNGERLEIDER and MISHKIN, 1982), is that the visual cortical areas are arranged into two visual processing pathways: a ventral pathway for perception and a dorsal pathway for action (GOODALE and MILNER, 1992, MILNER and GOODALE, 2006). However, recent work has started to question the validity of this influential model, stating that it might be too strong and inconsistent with recent data (DE HAAN and COWEY, 2011, SCHENK and MCINTOSH, 2010). In particular, it is increasingly unclear if the human visual cortical system is comprised of just two or more visual pathways, or none at all. Indeed, the primary support of the dual pathway model in humans has been deduced from dissociable consequences of naturally occurring lesions and functional localisation studies (GOODALE and MILNER, 1992, HAXBY et al., 1991, MILNER and GOODALE, 2006, UNGERLEIDER and HAXBY, 1994). Yet, although these studies inform about the distribution of dissociable functions across visual cortex, they do not demonstrate the existence of interconnected pathways nor can they rule out the possibility of additional cortical visual pathways.

There are several reasons why additional processing pathways might be expected in humans. First, the human brain, both in terms of its overall volume and the size of visual cortex, is much bigger than the primate brain, suggesting additional functions that might require additional or more specialised visual processing capabilities. Second, previous work suggests the existence of a large white matter fibre bundle, the inferior fronto-occipital fasciculus connecting human ventral occipital and inferior frontal cortex, that appears absent in non-human primates (CATANI and THIEBAUT DE SCHOTTEN, 2008, FORKEL et al., 2014). Third, recent theoretical work proposed the existence of three major visual processing pathways in humans. For instance, based on observations of multiple clusters of face- and limb-selective regions on the lateral and ventral occipitotemporal surfaces of the brain, it has been proposed that the human cortical visual system comprises a dorsal occipitoparietal stream, a lateral occipitotemporal stream and a ventral occipitotemporal stream (Weiner & Grill-Spector, 2013). In this model, the additional lateral stream, consisting of areas that are classically assigned to the dorsal stream, incorporates different aspects of vision, action and language. This view expands on earlier proposals that the difference between humans and non-human primates in the anatomical location of area MT/V5 relative to other dorsal and ventral visual areas might be related to a cortical expansion to accommodate language function in humans (ORBAN et al., 2004, UNGERLEIDER et al., 1998). The proposal of separable visual processing streams in human lateral and ventral occipitotemporal cortex also fits well with data suggesting a duplication of various other types of object response-selectivity across these two pieces of cortex (HASSON et al., 2002, KONKLE and CARAMAZZA, 2013, TAYLOR and DOWNING, 2011).

Despite an increasing appreciation of the similarity of the retinotopic organisation of occipital cortex in humans and non-human primates, there further are salient differences in the relative position of several high-level visual areas (ARCARO and KASTNER, 2015, ORBAN, 2016, ORBAN et al., 2014, VANDUFFEL et al., 2014). For instance, recent measurements suggest that areas LO-1 and LO-2, located on the lateral occipitotemporal surface of the human brain, have undergone a large-scale relative location-shift with respect to their putative homologues, V2A and OTd, in the macaque. V4A and OTd are located directly adjacent to V4v and inferior to the MT/V5 cluster and are part of a cluster of posterior inferior temporal (PIT) areas. Human areas LO-1 and LO-2 are located more superiorly, directly adjacent to V3d and they appear to be disconnected from the putative human PIT (phPIT) cluster. In addition, the human hemifield representation hV4 can be found on the ventral occipitotemporal surface, whereas its putative homologue in the macaque is comprised of a ventral upper quadrant representation and a lateral lower quadrant representation of the visual field. In humans there further exist visual field maps on the ventromedial occipitotemporal surface, VO-1 and VO-2 (BREWER et al., 2005, WANDELL et al., 2007). These areas might correspond to cytoarchitectonic area TFO in the macaque, for which recent preliminary evidence suggests that it consists of two central visual field maps that have been tentatively labelled TFO-1 and TFO-2 (Orban et al., 2014). In the macaque, TFO-1 directly abuts area V4A, while their putative human homologues, VO-1 and LO-1, are separated by area hV4 (several centimetres of cortex). These differences in the topological arrangement of some of the high-level retinotopic areas suggest a large-scale reorganisation that appears consistent with the notion of an additional lateral pathway in humans (Weiner & Grill-Spector, 2013), though it would suggest that the pathway originated from the classical ventral stream.

In addition to these considerations, still other theoretical accounts propose that the classical ventral and dorsal visual pathways should not be understood as unified systems. For instance, Kravitz et al. (KRAVITZ et al., 2011, KRAVITZ et al., 2013) proposed the existence of three dorsal pathways for visuospatial processing related to spatial working memory, visually guided attention and navigation, and six ventral sub-systems that each serve specialised behavioural, cognitive and affective functions. Importantly, this work relates to the macaque and thus argues that the classical dual-systems hypothesis has been over-simplified from its outset. This would suggest that the human visual system should also not be understood in terms of two (and only two) unified visual processing pathways. However, it is also possible that the apparently distinct functional properties within pathways do not reflect strictly separate sub-pathways, but a gradient-like organisation (Freud, Plaut, & Behrmann, 2016).

All these theories notwithstanding, the notion of two visual processing pathways ultimately concerns an empirical hypothesis about the wiring of the cortical visual system. Thus, it must be tested against the connections between the cortical visual areas. Indeed, the work that led to the postulation of the dual-pathways hypothesis involved a series of cross-lesion disconnection studies (MISHKIN, 1966, UNGERLEIDER and MISHKIN, 1982). These studies cannot be performed in humans, because they involve removing cortical areas as well as interhemispheric connections. Additional connectivity-based evidence for two visual pathways in non-human primates was presented in the early nineties, which was based on anatomical tract tracer injection data. In their seminal work, Felleman and van Essen presented a matrix of the connections between the visual areas of the macaque (Felleman & Van Essen, 1991). The availability of this matrix enabled Young to derive the topological organisation of the cortical visual connectome in a data-driven manner and confirm that the structural connectivity markers can be grouped along two separate visual processing pathways (Young, 1992). Young's approach is agnostic to the functional relevance of the putative pathways but can be applied in humans, provided that a similar connection matrix can be obtained non-invasively. In the present work, therefore, we set out to derive a connection matrix for the human cortical visual system and test by Young's approach whether human visual cortex comprises similar processing pathways as the macaque.

Anatomical tract tracer injection studies are invasive and therefore not applicable to humans. White matter tractography based on diffusion imaging is applicable to humans, but it is yet too limited in its ability to accurately determine a tract's cortical endpoints, particularly within a system that involves a highly dense network of many crossing, U-shaped and trans-callosal fibres (JBABDI and JOHANSEN-BERG, 2011, JBABDI et al., 2015). We therefore elected to characterise the patterns of connectivity between the human cortical visual areas directly at each cortical location based on correlated spontaneous blood oxygen-level dependent (BOLD) signal fluctuations that occur at rest. Known as resting-state fMRI (Fox & Raichle, 2007), this technique principally offers a measure of function rather than anatomy, though it has been shown to adhere closely to anatomical connectivity as derived from anatomical tract tracer injections (JBABDI et al., 2015, VINCENT et al., 2007, WANG et al., 2013). (And insofar differences exist, it would seem more appropriate to test a hypothesis about the functional organisation of the brain based on a measure of function rather than anatomy.) Resting-state fMRI also provides a natural measure of the connectivity strength. Once the putative pathways have been identified by Young's approach, this feature offers the opportunity to quantitatively confirm their existence by testing the prediction that the connections within a pathway are stronger than between different pathways (De Haan & Cowey, 2011).

## 2 Methods and materials

### 2.1 Data and pre-processing

We based our analyses on the publicly available 3T resting-state fMRI data acquired as part of the WU-Minn Human Connectome Project (SMITH et al., 2013, VAN ESSEN et al., 2013). The dataset concerned data-release S500, which contained 511 subjects in total, 470 of whom completed all resting-state fMRI runs. Two 14.4 min multi-band accelerated resting-state scans were recorded on each session day (TR = .72 sec; 2^3^mm^3^ isotropic voxels). The resting-state fMRI datasets were pre-processed as detailed in (Smith et al., 2013), which involved corrections for spatial distortions and head motion, registration to the T1w structural image, resampling to 2 mm MNI space, global intensity corrections, high-pass filtering with a cut-off at 2000 sec, and the ICA+FIX artefact removal procedure (GRIFFANTI et al., 2014, SALIMI-KHORSHIDI et al., 2014). We additionally spatially smoothed the data using a 3 mm FWHM isotropic Gaussian kernel and normalised the within-run data to zero mean and unit variance over time before concatenating the fMRI runs into one 28.8 min dataset for each session day.

### 2.2 Regions-of-interest

Visual areas were defined using a probabilistic atlas in MNI space of 50 retinotopic maps (25 in each cerebral hemisphere; though the atlas is in MNI space, the areas were defined on a reconstruction of the cortical gray-matter surface) (Wang, Mruczek, Arcaro, & Kastner, 2015). The retinotopic maps covered the following visual areas: V1 (primary visual cortex), V2, V3, hV4, VO-1/2, PHC-1/2, V3A/B, V7 (IPS-0), IPS1-5, SPL-1, FEF, LO-1/2, TO-1/2 (V5/MT+). See Wang et al. (2015) for a visualisation of the anatomical locations of these areas. The probabilistic atlas maps were trimmed into non-overlapping ROIs to minimise the effects of BOLD signal contamination, which is known to deteriorate network estimation quality (Smith et al., 2011). This was done by selecting only those voxels with an area membership probability that exceeded the top 99% of the robust range (i.e., within 2% and 98%) of all membership probabilities for that particular visual area across all voxels in the brain. This resulted in non-overlapping, gray-matter confined area definitions of at least 24 voxels (192 mm^3^) and separated by at least 2 mm. It is of note that applying a single threshold for all areas did not result in adequate trimming because the range of area membership probability varies across regions and therefore yielded either zero voxels for some areas (empty ROIs) or voxels that were not uniquely assigned to a single area (overlapping ROIs). The ensuing probably maps where next binarised and down-sampled from 1 mm to 2 mm isotropic resolution using nearest-neighbour interpolation. We then extracted the mean time-series (across voxels) from each visual area in each HCP subject.

### 2.3 Partial correlation analysis

Having obtained the mean time-series for each visual area and HCP subject, we next conducted a partial correlation analysis, computing the Pearson correlation coefficient (across the temporal domain; 2400 time-points) between each pair of areas while controlling for the activity in all other areas (i.e., regressing out the 48 mean time-series of the other visual areas and computing the correlation between the residuals). Compared with full correlations, this provides more accurate estimates of direct connectivity strength (Smith et al., 2011), but qualitatively similar results could also be obtained based on full correlations (Supplementary Fig. 3). Finally, the ensuing 50 × 50 connection matrices were stored for each subject and session day for subsequent analysis in Matlab (Mathworks, Natick MA).

### 2.4 Multi-dimensional scaling

Non-classical multi-dimensional scaling (MDS) was performed with Kruskal's normalised stress1 as the criterion to minimise (function ‘mdscale’ in Matlab). The dimensionality of the ensuing embedding was set to 2. MDS was applied separately to the group-level connection matrices associated with each session day. To obtain these group-level connection matrices, subject-level partial correlations were *z*-transformed, averaged across subjects, back-transformed to correlation values and then transformed into distances using the cosine theorem $\left(d=\sqrt{2\left(1-r\right)}\right)$ (Gower & Legendre, 1986).

We determined the dimensionality of the MDS embedding by assessing its reproducibility across session days (i.e., the variance explained as determined by Procrustes analysis) for 1–10 embedding dimensions. When the group-level connection matrix of 100 unrelated subjects was submitted to this analysis, the reproducibility across session days peaked when the dimensionality was set to 2. However, when the connection matrix of all 470 subjects was submitted, the reproducibility was at ceiling (*R*^2^ > .99) for dimensionalities 1–4. Therefore, we also performed a split-half cross-validation analysis by averaging all 470 subject-level connection matrices across the two session days and then running through 1000 iterations of randomly assigning half of the subjects to group A and the other half to group B. Within each iteration, the connection matrices where averaged across subjects to produce two group-level connection matrices (one for group A and one for group B), which were then submitted to MDS with varying dimensionality (1–10) and compared by Procrustes rotation. This confirmed that the two-dimensional MDS embedding exhibited the greatest reproducibility (*R*^2^ = .991).

### 2.5 Statistical analyses

To avoid possible biases due to the family structure of the HCP data (subjects were drawn from a population of twins and their non-twin siblings) (Van Essen et al., 2013), we restricted group-level statistical hypothesis testing to a subsample of 100 unrelated subjects. These subjects corresponded to those included in the “100 unrelated subjects” dataset available at db.humanconnectome.org. The tests involved paired-sample, two-sided *t*-tests between the subject-level within-stream and between-stream connectivity strengths for all of the three possible pairs of streams. Within-stream connectivity strength was defined as the average *z*-transformed partial correlation ($z=\text{atanh}\left(r\right)\sqrt{n-3}$, where *n* is the number of time points) across all within-stream connections (i.e., all connections within one stream, combined with all connections within a second stream).

Between-stream connectivity strength was defined as the average *z*-transformed partial correlation across all connections between areas that belonged to either of the two visual processing streams under consideration. Visual areas were assigned to the putative ventral stream if they are located on the ventral surface of the occipitotemporal lobe, to the putative lateral stream if they are located on the lateral surface of the occipitotemporal lobe, and to the dorsal stream if they are located on the dorsal surface of the occipitoparietal lobe (WANDELL et al., 2007, WANG et al., 2015). Early visual areas V1, V2 and V3 were deemed not to belong to any particular stream and therefore excluded from these comparisons. Reported effect sizes correspond to Cohen's *d* for repeated measures ($d_{rm}=t\sqrt{2\left(1-r\right)/n}$, where *t* is the *t*-statistic, *r* the correlation between the within- and between-stream connection strengths, and *n* the number of subjects) (Dunlap, Cortina, Vaslow, Burke, 1996).

To additionally determine whether the connectional separation between streams could also be observed at the level of single subjects, we compared the (un-averaged, *z*-transformed) within-stream and between-stream connections for each pair of streams and both session days within each of the 470 HCP subjects using two-sided, unpaired *t*-test (un-equal variances assumed). We then counted the number of subjects that exhibited significantly greater within-stream connection strength for all comparisons (i.e., the number of subjects that exhibited a significant effect for all pairs of streams and both session days).

### 2.6 Hierarchical clustering analysis

We additionally determined the number of streams using a connectivity-based hierarchical cluster analysis. Given the almost perfect reproducibility of the MDS results across session days (*R*^2^ > .99), we first averaged the group-level connection matrices across session days and submitted the ensuing matrix to the MDS procedure described above. We then converted the ensuing embedding (excluding early visual areas V1-3) into a connected graph and fed it to Ward clustering (function ‘linkage’ in Matlab). The conversion into a graph was based on a *k*-nearest neighbour search (function ‘knnsearch’ in Matlab) using the Euclidean distances between the points in the embedding. The parameter *k* was set to the minimal value (i.e., *k* = 4) that produced a graph with a single connected component. We computed the cophenetic correlation coefficient (*c* = .71) to verify that the ensuing dendogram faithfully represented the configuration given by the MDS embedding (function ‘cophenet’ in Matlab). To determine the optimal number of clusters, we examined the effect size $\left(d_{\text{rm}}\right)$ of the difference between all within-cluster and all between-cluster connectivity strengths while increasing the number of clusters.

## 3 Results

In much the same way as Young demonstrated the existence of two cortical visual pathways in the macaque (Young, 1992), we used multidimensional scaling (MDS) to analyse a matrix of connections between the cortical visual areas (see Supplementary Fig. 1). Here, the connection matrix was derived by extracting, for each of 470 Human Connectome Project (HCP) subjects, the mean resting-state fMRI time courses from the visual areas and then performing a partial correlation analysis to estimate the connectivity strength between each pair of them. The visual areas were defined using a recently published probabilistic atlas, which offers the most comprehensive parcellation of human visual cortex to date based on independent retinotopic mapping data (Wang et al., 2015). It provides probabilistic maps of as many as 50 visual field representations (retinotopic maps) covering a total of 22 distinct visual areas. The dimensionality of the MDS embedding was determined by assessing its reproducibility across session days, which, in agreement with Young (Young, 1992), was maximal when set to two.

**Fig. 1:**
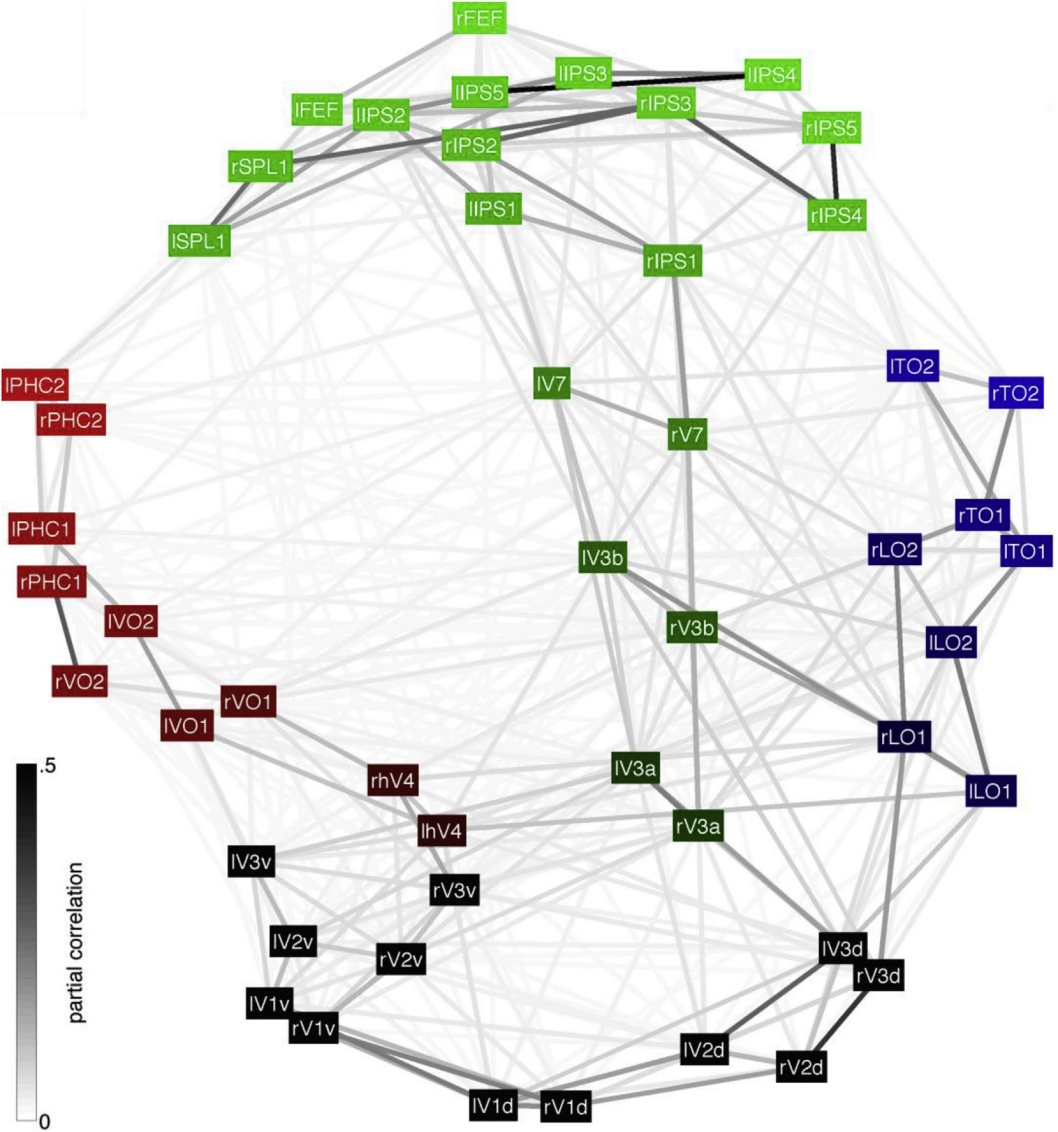
The topological organisation of the human cortical visual connectome. MDS results for session day 1 (see Supplementary Fig. 2 for the results of session day 2). Functional connections are colour-coded according to the group-level (*N* = 470) partial correlations (back-transformed from *z* to *r* values after averaging) between the mean resting-state fMRI time-series of each of the area-pairs. Area boxes are colour-coded according to their anatomical locations (WANDELL et al., 2007, WANG et al., 2015), with colour-intensity weighted by the distance to V1-3. Black boxes indicate early visual areas on the medial occipital surface (V1-3). Red boxes indicate areas on the ventral occipitotemporal surface (hV4, VO1/2, PHC1/2). Blue boxes indicate areas on the lateral occipitotemporal surface (LO1/2, TO1/2). Note that TO1/2 corresponds to V5/MT+; (Amano, Wandell, & Dumoulin, 2009). Green boxes indicate areas on the occipitoparietal (V3A/B, V7, IPS1-5, SPL1) and frontal cortices (FEF).

Fig. 1 shows the ensuing topological organisation of the human cortical visual connectome. This structure was highly reproducible across session days (*R*^2^ = .99, determined by Procrustes analysis; see Supplementary Fig. 2) and could not be derived based on the volumetric distances between the visual areas (*R*^2^ = .22) or the relative ROI sizes (*R*^2^ = .11; different ROI sizes may yield differences in the SNR of the mean signal extracted for each area). As in the macaque, the human cortical visual system appears to be largely hierarchically organized, with the strongest connections between neighbouring areas as well as homotopic locations in the opposing hemispheres throughout the visual hierarchy (see Table 1). Unlike in the macaque, our wiring diagram suggested not two but three visual processing pathways: one on the ventral occipitotemporal surface (red), one on the lateral occipitotemporal surface (blue), and one dorsal on the occipitoparietal surface (green).

**Table 1:** Quantitative comparisons between Fig. 1 and possible organisational models. Comparisons were performed by Procrustes rotation. The statistical significance (probability) of the associated variance-explained (*R*^2^) statistics was assessed by repeating the Procrustes rotation after randomly permuting the area labels of the numerical models on each of 100,000 iterations while noting the fraction of times that the variance-explained statistic exceeded or was equal to the variance explained by the un-permuted organisational model. The ‘nearest-neighbour’, ‘nearest-neighbour or next-door-but-one’, ‘interhemispheric’ and the ‘combined nearest-neighbour or next-door-but-one and interhemispheric’ models were constructed by creating artificial connectivity matrices and submitting these to the same MDS procedure that was used to derive the structure shown in Fig. 1. The ‘nearest-neighbour’ and ‘nearest-neighbour or next-door-but-one’ connectivity matrices were constructed as described in Young (1992). To construct the ‘interhemispheric’ connectivity matrix, we scored all connections between homologous areas in the opposing hemispheres as ‘1’ and all other connections as ‘0’. The ‘combined nearest-neighbour or next-door-but-one and interhemispheric’ connectivity matrix was defined as the sum of the ‘nearest-neighbour or next-door-but-one’ and ‘interhemispheric’ matrices. The hierarchical model was constructed as a one-dimensional vector of shortest path-lengths through the ‘nearest-neighbour’ matrix between each area and ipsilateral V1 (as determined by Dijkstra's algorithm). The two and three streams models were constructed as one-dimensional vectors of values representing each area's stream category based on the colour-coding in Fig. 1. Note that V1, V2 and V3 were excluded from these comparisons since these areas were deemed not to belong to any particular visual processing stream; they were excluded from all comparisons reported in this table to allow for comparison across models. Although all of the modelled organisational principles appear to be reflected in Fig. 1 to some extend, the topological organisation of the human visual connectome is most parsimoniously and fully explained by the ‘combined hierarchical and three streams’ model.

Organisational Model	*R*^2^	Probability
Nearest-neighbour	.14	*p* = .01
Nearest-neighbour or next-door-but-one	.13	*p* = .02
Interhemispheric	.08	*p* = .05
Combined nearest-neighbour or next-door-but-one and interhemispheric	.67	*p* < 10^−5^
Hierarchical	.35	*p* < 10^−5^
Three streams	.47	*p* < 10^−5^
Combined hierarchical and three streams	.80	*p* < 10^−5^
Combined hierarchical and two streams:		
ventral + lateral *vs* dorsal	.25	*p* < 10^−4^
dorsal + lateral *vs* ventral	.68	*p* < 10^−5^
ventral + dorsal *vs* lateral	.66	*p* < 10^−5^

**Fig. 2:**
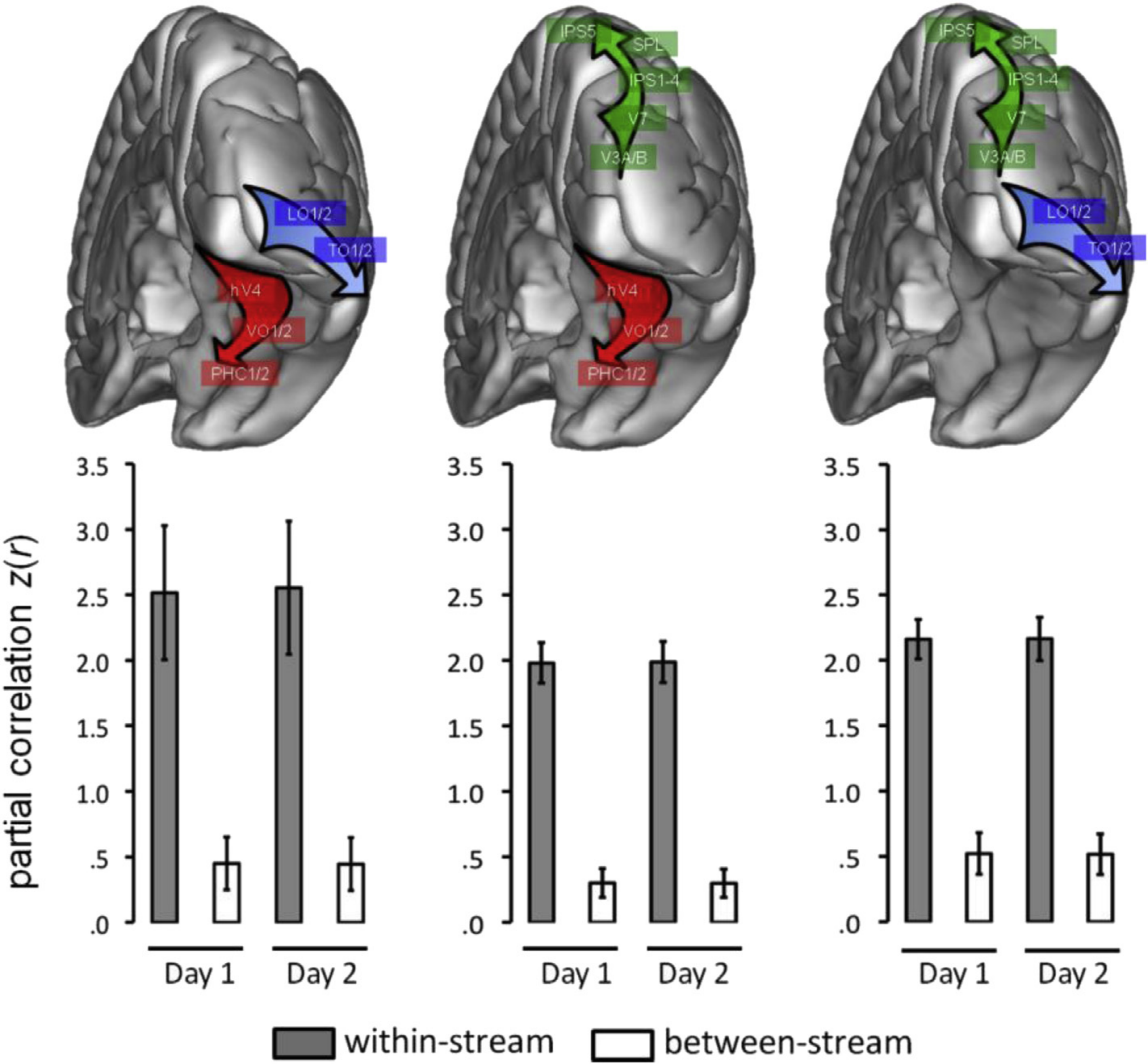
Triple dissociation between ventral, lateral and dorsal visual cortex. Panels show the average (*z*-transformed) between- and within-stream connectivity strengths across 100 unrelated subjects for the putative ventral and lateral streams (left) the putative ventral and dorsal streams (middle), and the putative lateral and dorsal streams (right). Error-bars indicate the standard deviation. Approximate visual area locations are indicated by transparent boxes on a three-dimensional rendering of the right cerebral hemisphere.

“A pathway model predicts that the connections between the maps that are within a pathway are significantly stronger than the connections between different pathways” (De Haan & Cowey, 2011). To quantify the evidence of three separable pathways, therefore, we computed the average connectivity strength within and between each of the three possible pairs of pathways suggested by the structure shown in Fig. 1. Accordingly, visual areas located on the ventral and lateral occipitotemporal cortical surfaces were assigned to the putative ventral and lateral pathways, and those on the occipitoparietal cortical surface were assigned to the putative dorsal pathway (see Fig. 2A). To avoid potential biases due to the family structure of the HCP data (Van Essen et al., 2013), we tested for a difference in within-pathway versus between-pathway connectivity strength in a subset of 100 unrelated subjects. This revealed highly significantly stronger within-pathway connectivity at the group-level for all pairs of pathways and both session days (all *t*_99_ ≥ 34.8, *p* = 0, *d*_rm_ ≥ 5.50). This triple dissociation could also be observed within single subjects as the within-pathway connections were significantly (*p* < .05) stronger than the between-pathway connections for all pairs of pathways and session days in 99 of the 100 unrelated and 465 of all 470 HCP subjects.

Despite the fact that we trimmed the probabilistic atlas maps into spatially segregated ROIs, it is likely that this approach did not completely eliminate the possible BOLD signal contamination (i.e., due to spatial smoothing and/or inter-subject variability) across neighbouring ROIs, which could have lead to inflated connectivity strength estimates the connections between areas and their direct neighbours. To investigate the extent to which this might have influenced the configuration shown in Fig. 1, we compared it against a configuration with reduced nearest-neighbour connections. Specifically, a reduced connectivity matrix was set-up by setting all connections between neighbouring areas to 1/3th their original value.^1^ This reduced connectivity matrix was then fed to the same procedure used to derive the original configuration, after which both structures were compared by Procrustes rotation. This model explained 99.87% and 99.80% of the variance in the original configuration for session days 1 and 2, respectively, indicating that possible BOLD signal contamination has had no substantial influence on the findings. In line with this, highly significantly greater within-pathway connectivity strengths could still be observed for all pairs of pathways at the group-level (all *t*_99_ ≥ 22.7, *p* = 0, *d*_rm_ ≥ 3.62) as well as within single subjects (*p* < .05 for all pathway-pairs and session days in 77% of the subjects).

**Fig. 3:**
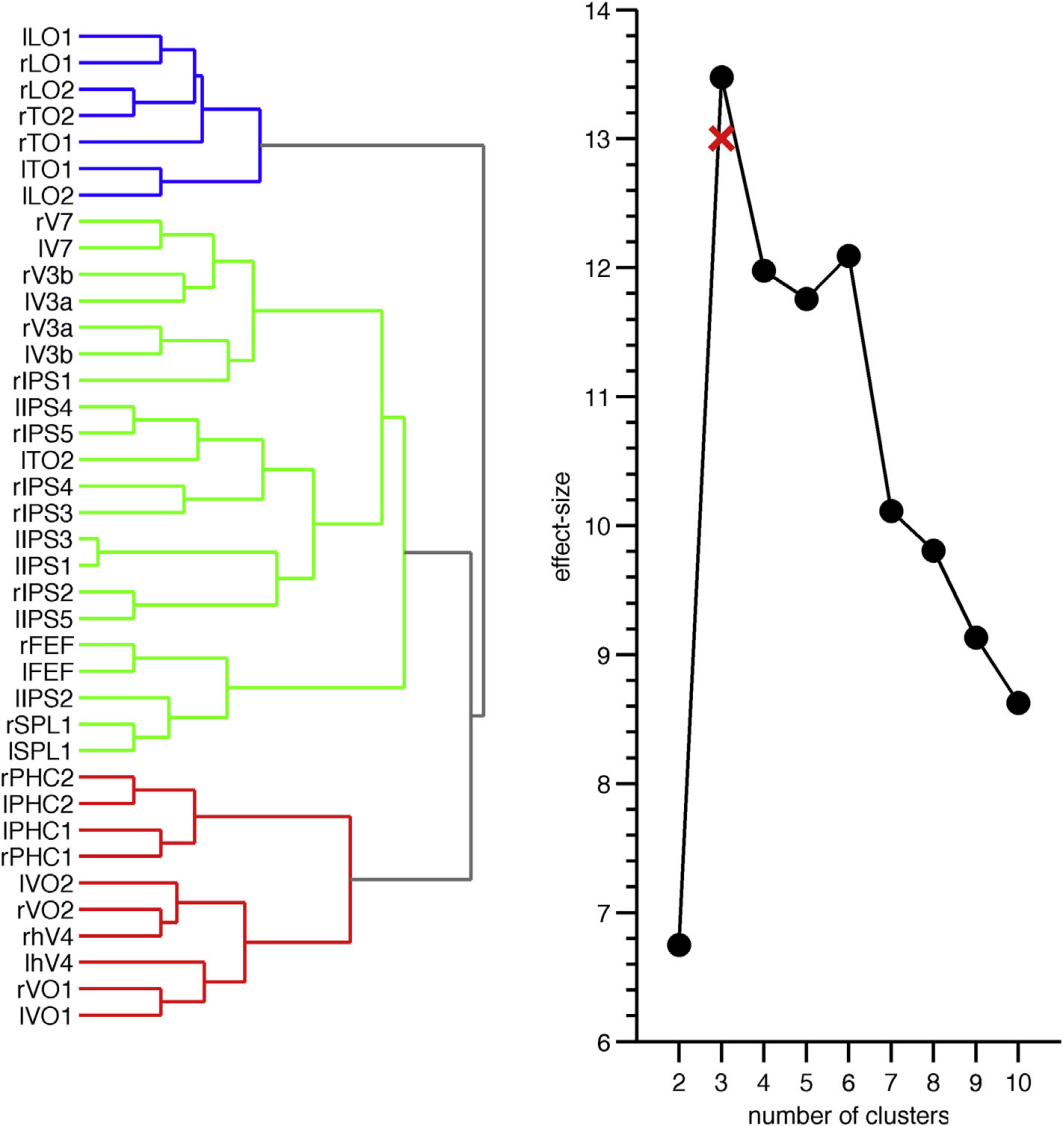
**Hierarchical clustering results.** The optimal number of clusters is given by the maximal effect size (Cohen's *d* for repeated measures; *N* = 470) of the difference between the within-cluster and between-cluster connectivity strengths (i.e., the partial correlation between area pairs as shown in Fig. S1). The red ‘x’ in the right panel indicates the corresponding effect-size (*N* = 470; all within-stream versus all between-stream connections, both session days combined) for the area-to-stream assignments based on their anatomical locations (see Fig. 2), which, except for area lTO2, were identical to the optimal hierarchical clustering results (*k* = 3; left panel).

The triple dissociation indicates that we can identify at least three separable cortical functional pathways. To determine whether there might be more than three pathways, we applied a hierarchical clustering analysis to the configuration shown in Fig. 1. This revealed that the difference between the within- and between-pathway connection strengths is maximised for three pathways, with nearly identical area-to-pathway assignments as before (Fig. 3). Thus, the functional organisation of the human cortical visual system is best characterised as being comprised of three visual pathways.

## 4 Discussion

We characterised the topological organisation of the human cortical visual connectome based on resting-state fMRI connectivity estimates in 470 individuals. The ensuing configuration was highly reproducible and appears to be inherently two-dimensional, with one axis of organisation indicating a distinction between three separate visual pathways (along the horizontal axis), and the other suggesting hierarchical organisation (along the vertical axis). The distinction between three visual pathways was confirmed quantitatively by a highly significant triple dissociation in within-versus between-pathway functional connectivity strength that could be observed across sessions in more than 99% of the subjects. Young, who pioneered this MDS approach in the macaque, observed a similar two-dimensional topological organisation, whilst indicating just two visual pathways (Young, 1992). This discrepancy may be due to differences between species or methodology: Young based his analyses on anatomical tract tracing data in the macaque, whereas we here used resting-state functional MRI connectivity in humans. However, previous work in non-human primates has demonstrated that resting-state fMRI connectivity adheres closely to anatomical connectivity estimates based on tract tracer injections (JBABDI et al., 2015, VINCENT et al., 2007, WANG et al., 2013). Thus, it appears unlikely that the discrepancy is due to differences between functional and anatomical connectivity per se. However, we cannot rule out that the triple dissociation might be strictly functional.

Another, more subtle, methodological difference involves the usage of discrete versus continuous connectivity information. Young based his analysis on a matrix coding only the presence or absence of unidirectional and bidirectional connections (Young, 1992), whereas ours coded a continuous scale of connectivity strengths (partial correlations). It is therefore possible that the discrepancy does not reflect a difference between species, but that our connection matrix carried information necessary to distinguish the three pathways that was not available to the previous work in the macaque. This could mean that the macaque cortical visual system also comprises three visual pathways (but not that humans have two pathways, since information was added). In addition, though Felleman and Van Essen (Felleman & Van Essen, 1991) accumulated extensive data on the anatomical connectivity of the macaque visual system, they acknowledged that there is a fair amount of uncertainty in their connectivity matrix, which may have influenced Young's result. That said, Young's result is corroborated by years of preceding research and it is known that extrastriate cortex is greatly expanded in humans, with a large white-matter fibre tract that does not have a clear homologue in the macaque (CATANI and THIEBAUT DE SCHOTTEN, 2008, FORKEL et al., 2014, ORBAN et al., 2004, VAN ESSEN et al., 2001, WANDELL et al., 2007). Moreover, there are clear differences in the topological arrangement of homologous visual areas in humans and non-human primates (ORBAN et al., 2014, VANDUFFEL et al., 2014), suggesting a large-scale reorganisation of the overall functional architecture of the visual cortical system. Thus, the most parsimonious explanation for the discrepancy is that, since the time of phylogenetic separation from the macaque 28-25 million years ago (Rogers & Gibbs, 2014), the human lineage has evolved to comprise not two but three visual pathways.

Although the probabilistic atlas used here involves the most comprehensive and precise atlas of the human cortical visual areas to date, it does not include known visual areas with visual field maps of the peripheral visual field (Wang et al., 2015). Therefore, areas such as V6 and V6A could not be included in our analyses. Omitting these and potentially other currently unreported visual field maps leads to an over-estimation of the connectivity between areas that are possibly indirectly connected via these missing nodes because the activity of the missing nodes has not been partialled out. Such over-estimation would be most profound for connections between areas that are not directly adjacent. Thus, omitting areas would primarily lead to inflated between-pathway connectivity strength estimates, and therefore an under-estimation of the true difference between within-pathway and between-pathway connectivity strengths. Therefore, we believe that our results are a conservative under-estimation of the true segregation between the three functional pathways described in this work. It remains possible, however, that additional pathways are discovered composed of visual areas, such as for instance putative human PIT (KOLSTER et al., 2010, ORBAN et al., 2014), that have not been included in the atlas—and therefore do not appear in our analyses—perhaps because the evidence of their existence was considered too preliminary.

In line with Young's result (Young, 1992), one dimension of the MDS embedding appears to reflect hierarchical organisation. Young was able to formally test for hierarchical organisation by comparing the embedding against a hierarchical ladder based on the laminar origin and termination patterns of the connections in the macaque (Felleman & Van Essen, 1991). Such information is not yet available in humans, and so we here constructed a hierarchical ladder based on the shortest paths through a nearest-neighbour graph (i.e., a graph with edges between direct neighbours only) between each area and V1 (Table 1). It is also possible to informally compare the hierarchical organisation suggested by the present results with the hierarchical organisation suggested by the neuronal receptive field sizes that have been reported for several (but not yet all) human cortical visual areas (Wandell & Winawer, 2015). The neuronal receptive field size is known to increase up the visual hierarchy due to spatial pooling from upstream areas (HAAK et al., 2013, HARVEY and DUMOULIN, 2011, MOTTER, 2009, PERRETT and ORAM, 1993, RIESENHUBER and POGGIO, 1999, TANAKA et al., 1991, WANDELL and WINAWER, 2015), and it might thus be expected that the ordering of the areas within each of the pathways along the vertical dimension of the MDS embedding adheres to the ordering of the receptive field sizes reported for those areas (Ungerleider & Haxby, 1994). Indeed, within each pathway, the ordering of the visual areas along the vertical axis of the MDS embedding is a perfect predictor of the ranked ordering of the human neuronal receptive field sizes that have been estimated to date (Wandell & Winawer, 2015). Thus, insofar as the neuronal receptive field size can be taken as an indicator of an area's position in the visual hierarchy, the human visual system appears to be organised into three hierarchically organised processing pathways.

Interestingly, also, the human MDS projects lateral regions farther away from ventral regions than dorsal regions. This implies that the connectivity ‘fingerprints’ of lateral regions are more similar to those of dorsal regions than they are similar to the connectivity fingerprints of ventral regions. This, in turn, suggests that lateral stream function is more similar to the dorsal stream than the ventral stream (Passingham, Stephan, & Kotter, 2002), which would be consistent with the theoretical proposal that human visual cortex comprises a third lateral stream that has evolved to become detached from the classical dorsal stream to accommodate language function in humans (Weiner & Grill-Spector, 2013). Indeed, the lateral pathway in humans includes areas TO-1 and TO-2 (MT/V5), which are traditionally associated with the dorsal pathway. The ordering of streams along the horizontal axis of the human embedding further indicates that it does not reflect cortical distance in a straightforward way. This last point can also be taken from the fact that corresponding areas in the opposing hemispheres are consistently mapped close together throughout the MDS embedding.

The multiple pathways model (and recent refinements thereof) exists among several proposals about the principles that drive the organisation of the human cortical visual areas (Wandell et al., 2007). These models are not necessarily mutually exclusive. Indeed, it is quite possible that overlying modes of organisation exist, just as retinotopic representations (eccentricity and polar angle) and hypercolumns coexist within the visual areas. Young's and our MDS results both indicate that the principal modes of organisation reflect the distinct pathways and hierarchical processing, with little evidence of additional modes of organisation (in both humans and non-human primates the optimal MDS embedding dimensionality was two). This suggests that alternative proposals—if true—effectively describe the same dimension(s) as reported here. For instance, it has been proposed that visual cortex is arranged according to visual field map clusters (Wandell, Brewer, & Dougherty, 2005), which is compatible with the three pathways identified here because the proposed clusters would exist at a smaller scale (within pathways). The proposed clustering is not evident in the inter-areal connectivity data presented here but may well be evident from finer-grained characterisations. Note that a similar argument applies to the theoretical models of Kravitz et al. (KRAVITZ et al., 2011, KRAVITZ et al., 2013), who proposed that the classical dorsal and ventral streams should be understood as being comprised of several sub-systems.

The notion of separate visual pathways on the lateral and ventral occipitotemporal surfaces is also consistent with observations of a large-scale mirror-symmetric organisation of object response-selectivity. That is, the object-selective areas come in pairs, with one area on the ventral and another on the lateral occipitotemporal cortical surface (HASSON et al., 2002, KONKLE and CARAMAZZA, 2013, TAYLOR and DOWNING, 2011). On each side of the line of reflection, object response-selectivity (e.g., to faces, objects and buildings) appears to follow a centre-periphery organisation, while areas on the lateral and ventral occipitotemporal surface (superior and inferior to the line of reflection) exhibit biases toward processing the lower and upper quadrants of the contralateral visual field, respectively (Silson et al., 2013), and a differential involvement in attentive versus ambient viewing behaviour (Marsman, Renken, Haak, & Cornelissen, 2013). This could mean that the ventral and lateral pathways may be specialised for processing objects that appear in extrapersonal versus peripersonal visual space, which may have emerged as our ancestors started to walk on two feet.

At the current scale of description, however, our results appear most consistent with the model proposed by Weiner & Grill-Spector (Weiner & Grill-Spector, 2013). Indeed, this model proposed not only the existence of an additional lateral pathway, but also that the lateral pathway would be functionally more similar to the classical dorsal than the ventral pathway. The model suggests that the lateral pathway incorporates different aspects of vision, action and language. The idea that lateral visual object processing is related to the emergence of the human language faculty finds support in recent studies suggesting that the apparently uniquely human inferior fronto-occipital fasciculus (CATANI and THIEBAUT DE SCHOTTEN, 2008, FORKEL et al., 2014) subserves language semantics (Almairac et al., 2015, DUFFAU et al., 2013, HAN et al., 2013).

## 5 Conclusion

In the present work, we tested the inter-areal connectivity of the human cortical visual system against the classical idea that it is comprised of two distinct visual pathways. In line with recent theoretical proposals, we found that the human visual cortical areas are organised into not two but three visual pathways: one dorsal, one lateral and one ventral. It is likely that the additional lateral pathway is uniquely human, related to emergent language function. It appears, therefore, that the field attempted to fit strict functional dichotomisations onto an inherently tripartite organisation, which may explain at least some of the increasing controversy regarding the biological validity and functional interpretation of the classical dual-pathway model in humans.

## Footnotes

1 This down-weighting factor yields a conservative estimate of the true connectivity strength were it inflated due to BOLD signal contamination: for the smallest ROI, right VO1, which is expected to be most susceptible to BOLD signal smearing, it reduces the nearest-neighbour connectivity strengths to ∼60% of that area's interhemispheric connectivity with left VO1 (interhemispheric connectivity estimates should be free of BOLD signal contamination); across all ROIs it reduces the nearest-neighbour connectivity strength to ∼50% of the interhemispheric connectivity strength, on average.
