# Supplementary material for: Objective analysis of the topological organization of the human cortical visual connectome suggests three visual pathways
Source: Cortex. 2018 Jan;98:73–83. doi: 10.1016/j.cortex.2017.03.020 (PMC5780302; doi:10.1016/j.cortex.2017.03.020)
Supplement: Supplementary file 1 [file mmc1.pdf]

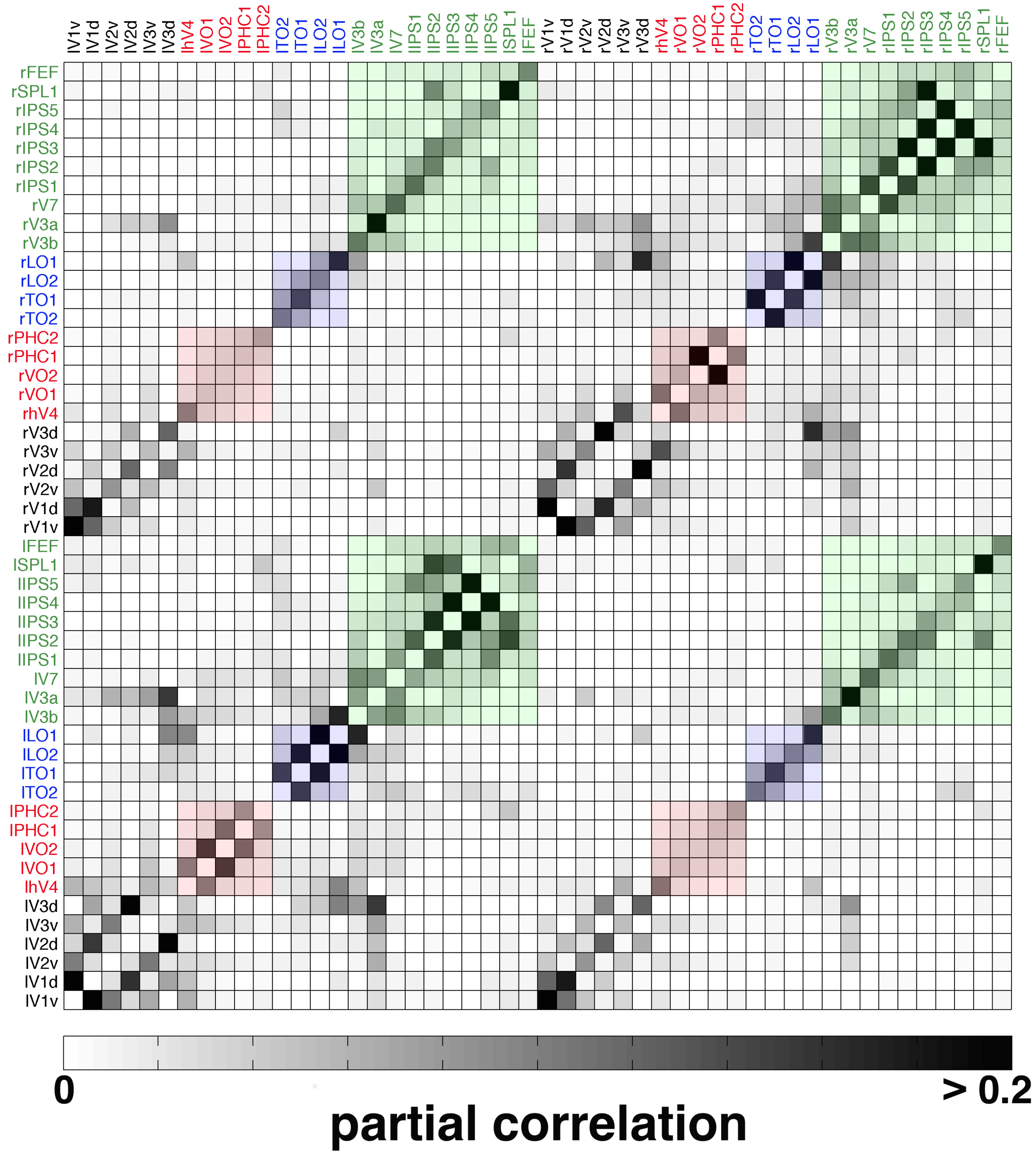

Supplementary Figure 1. Functional connection matrices for session days 1 and 2. Partial correlations (back-transformed to *r* values after averaging) between all pairs of visual areas from session day 1 are shown in the left upper-triangle and those from session day 2 are shown in the right lower-triangle. Area labels and transparent overlays indicating within-stream connections are coloured according to **Figure 1**.

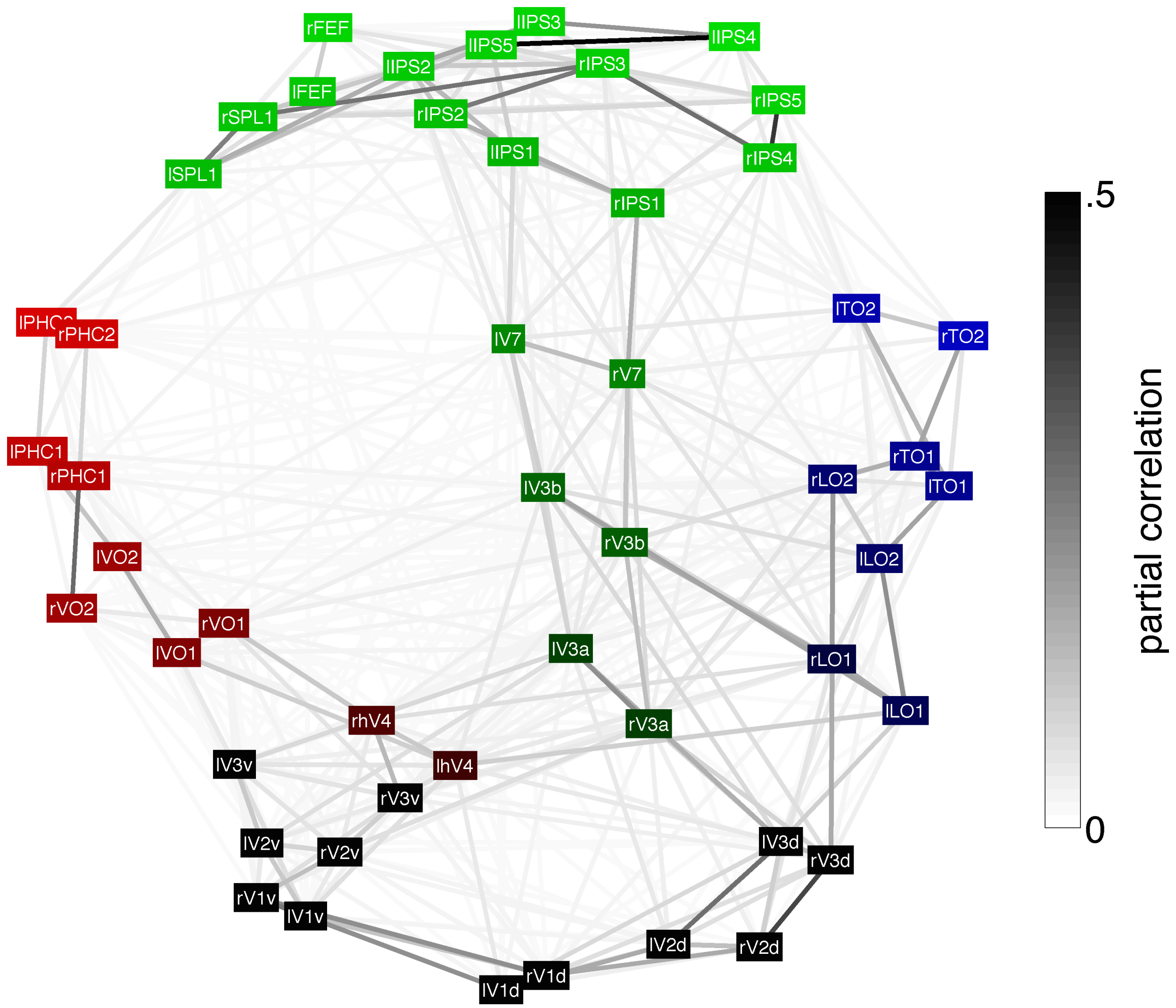

Supplementary Figure 2. MDS results based on the rfMRI data acquired during session day 2 (N=470). Conventions are as in main **Figure 1**.

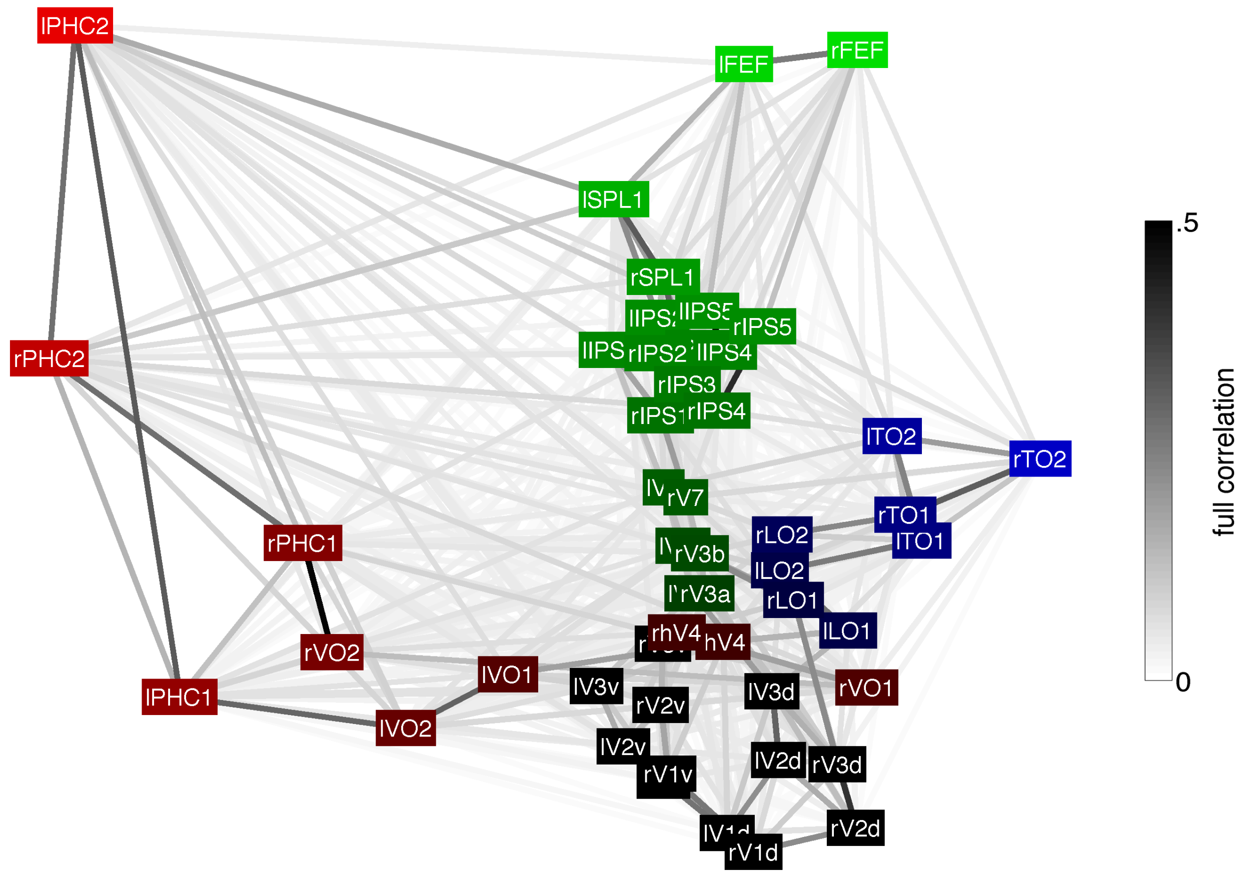

Supplementary Figure 3. MDS results based on full correlations using the rfMRI data acquired during session day 2 (N=470). Conventions are as in main **Figure 1** and **Supplementary Figure 2**. The MDS based on full correlations explained 71% of the variance in the MDS based on partial correlations and shows the same qualitative pattern of three visual pathways (one ventral, one dorsal, and one lateral). Quantitative differences can be attributed to the fact that full correlations cannot distinguish direct from indirect connections, leading to many more false positives than partial correlations. Nevertheless, just like the connectivity strength estimates based on partial correlations, the connectivity estimates based on full correlations were significantly stronger than the between-pathway connections (all $t_{99} = 17.7$, $p = 0$).
